# A Case of Railway Spine

**Published:** 1893-08

**Authors:** Allen J. Smith

**Affiliations:** Galveston, Professor of Pathology and Lecturer on Mental Diseases in the Medical Department of the University of Texas


### TEXAS MEDICAL JOURNAL.

ESTABLISHED JULY, 1885.

PUBLISHED MONTHLY.—SUBSCRIPTION $2.00 A YEAR.

Vol. IX. AUSTIN, AUGUST, 1893. No. 2.

### Original Contributions.

For Texas Medical Journal.

### A CASE OF RAILWAY SPINE.

ALLEN J. SMITH, M. D., GALVESTON,

Professor of Pathology and Lecturer on Mental Diseases in the Medical Department of the University of Texas.

THE terms "traumatic hysteria," "railway spine," "chronic spinal concussion," "traumatic neurasthenia," have all been applied to a condition, the basis of which is apparently largely but a neurosis, and in which there has failed as yet of demonstration a distinct material pathological foundation. The comparative newness and paucity of our knowledge of the affection may be invoked as an atoning explanation for the more or less general belief that the condition is without organic alteration, and is essentially functional and psychical in its nature; and will likewise, serve as sufficient reason for the addition of the following report to the literature of the subject.

*George H.*, a white man of thirty-seven years of age, married and a father, with parents, brother and sister living and in health, none of his immediate family dead, with no acknowledged neurotic taint in his family; with no discovered or acknowledged circumstance in his previous personal history bearing upon the point in question, a train employe of one of the railways of the State, asserts that on a certain date he was injured in a railway accident--an assertion not denied by the company interested in the litigation which has followed. The patient relates that standing upon the platform of a car at the time of the crash, he was thrown forward so as to receive a blow from the brake-wheel in the left mid-abdominal area. He was then thrown out from between the cars, landing some distance away, and striking the ground with his feet, in a semi-erect posture, then falling unconscious. He remained in this condition for perhaps half an hour, as estimated by himself afterwards, regaining his appreciation of his surroundings as he was being conveyd to a car for transportation to an adjacent station.

No history of his exact condition at the time has as yet been obtained by the writer, and the degree of the immediate shock is unknown to him. For two and a half months the patient asserts he was confined to his bed, unable to be about. As soon as possible, that is, within three months after the accident, he came to Galveston and entered Sealy hospital, and was placed in the surgical wards, where he remained several months, under treatment especially for an injury to the right knee received in the accident. The writer has no knowledge of the patient's condition during this period, the notes of the case having unfortunately been lost. After a time he left the hospital and came under the care of one of the physicians of Galveston until January 9, 1893, when he was readmitted to Sealy hospital and placed in the care of the neurological department, remaining under treatment in the wards for about a month. Since his departure from the hospital, the writer has had a number of opportunities to examine, and has had professional charge of the man. While he was in the hospital it was learned that immediately after the accident, the most marked symptom was pain, of general distribution, but worse in the limbs; and that he felt unable to move his legs because of the pain. The right patella was dislocated, according to his statement, and there was a slight flesh wound on the inner aspect of the left ankle. No bones were broken. Soon after the accident, after he had been carried into a house, he states that he spat blood; and that a pain located in the left hypochondrium gradually and progressively became worse, an accompanying tremor with spasm-like sensations developing in the muscles in this region. He vomited frequently after the accident, and describes an appearance of the vomited matter suggestive of the "coffee grounds" nature of altered blood. This symptom continued at intervals for about a year, until about Christmas, 1892, being generally worse immediately after eating. Eight or nine days after the accident he was seized for the first time with a hystero-epileptoid convulsion, and since then there have been numerous and distinct recurrences. The patient describes an aura preceding these seizures, of the nature of a globus, situated in the left hypochondrium. In these convulsions he acknowledges that he does not entirely lose consciousness, states that he hears and partially comprehends the words of those about him, and appreciates their acts; but denies that he is able to answer by word or act of his own. The convulsions are always unilateral, affecting the left side, and are of a clonic type, thus producing a hemi-choreoid movement. The face of the same side partakes in the convulsive movements. The patient complains of an almost constant headache, frontal, generally worse on the left side.

No signs of pulmonary, cardiac, gastric or renal disorders were detected upon examination, save on several occasions an undue rapidity of heart beats.

The man is of medium build, weighing perhaps 160 pounds, ruddy, with light hair and eyes; and would be classed by one not acquainted with his nervous history, as of a lymphatic temperament. The entire left side from head to foot, except a narrow band extending downward on the inner and posterior portion of the left thigh and about the anus, is distinctly though but partially anæsthetic, the opposite side being normal, and in few spots hyperæsthetic. The region excluded on the left side from the anæsthetic area corresponds fairly to that supplied by the two lower sacral nerves. Estimated by the grip and kick (the leg being held) the left side shows marked diminution of motor power also. In comparison with the right, the left side, particularly in the extremities, is cool to touch, and the left limb is not as large by actual measurement as its fellow. Along the spine there are on the left side tender spots, easily detected by pressure, or by application of heat, at the level of the fourth cervical, third or fourth dorsal and tenth dorsal vertebræ, as well as just above the sacrum; on the right side at the level of the eleventh dorsal vertebra. On the left breast in the lower and outer quarter is a similar spot. Deep pressure on these spots has never succeeded in inducing any other manifestation than that of intense pain and flushing of the face, with increase in frequency of the heart's action; and no relation has been demonstrated between them and the convulsive seizures. Heat applied over these spots causes localized muscular spasm as the hot sponge is drawn downward along the spine; and as each spot is passed the patient winces. The right knee is stiff, the supposed result of the patellar dislocation mentioned, and no reflex anomally can be detected. The left patellar reflex although present, is but slightly marked and probably diminished.

The sense of taste on the left side of the tongue is decidedly diminished, and for some things apparently perverted; is normal on the right side. Hearing on the left side is impaired, the watch heard normally at twenty inches being heard at only four or five; there is no evidence of external auditory disease. Similar as well as other aural disturbances are noted by Baginsky (*Berlin klin. Wochensch.* 3, 1888). The field of vison is contracted somewhat in both eyes; and the color fields show the curious irregularities occasionally found in cases of hysteria, particularly seen in the overlapping of the red and blue fields in the right eye. The visual contraction is more or less concentric, although in the left eye temporal rather than nasal. Retinal examination, kindly performed for the writer by Drs. Hodges and Haden of Galveston, (as too was the perimetry) was negative save for a small amount of pigment in the nasal side of the right eye. The color fields correspond with the writer's examination for colors by the ordinary color tests, in which the major colors were easily recognized but in which the secondary shade of blue and red were more or less mixed by the man. No differences in the pupils or other optic symptoms were noted. The activity of the sexual function and sexual desire were stated to be diminished. The want of proper apparatus prevented the examination of muscular tone.

Briefly summarized, the symptom complex of the case includes the following symptoms: Left sided hemi-anæsthesia, well marked, for touch, pain, heat and cold; motor paresis of the same side; occasional left-sided clonic convulsions of epileptiform; contraction of visual fields, especially on the temporal side of the left eye; left-sided partial deafness; a number of foci or tenderness along the spine, suggesting irritation of the emerging nerves, together with other symptoms.

It cannot be denied that such a group of phenomena is likely to be of hysterical origin, that from their subjective nature they are symptoms easily misjudged by the physician, and that they are even liable to be simulated with success by a designing person. Nor would it be in the least flattering to a physician to believe that he could overlook the strong element of hysteria present in the case narrated. Yet on the other hand, there are some things to be said upon the question of the existence, beyond and before all this, of a material fault, cellular or humeral, upon which, to some extent at least, these symptoms of traumatic hysteria depend. Non-belief in the existence of such a fault is to be deplored, as unlikely to ever permit a proper spirit of investigation as to the reality and demonstrability of the causal condition. The idea of a purely functional basis is most unwelcome to a morbid anatomist, whose realization is, that if no normal functions have as yet failed of anatomical solution, there is neither right nor reason in denying such marked alterations of the nervous functions an anatomical basis. Aside from the undeniable presence of a psychical element, the denial of an organic foundation for these symptoms rests almost entirely upon grounds of negation, upon the failure thus far of demonstration of any distinct lesion. Further, the apparent influence of pecuniary considerations in many cases, both upon the severity of the symptoms and their relief, has been often held as evidence of the improbability of the affection, a view which is favored by the subjective nature of the symptoms usually presented; such a view is, however, without much weight, inasmuch as the relief from worry occasioned by the adjustment of monetary claims must necessarily be a powerful factor in therapeusis. It must be remembered, however, that while much has been written upon the subject by clinical observers of no mean note and ability, in the rarely fatal nature of the affection little opportunity has been afforded to verify theories by direct and careful observation of possibly affected areas, and that until a further and goodly number of observations by competent pathologists have been recorded, one has no right to predicate the non-existence of material changes.

In the case reported by Kronthal and Sperling, the patient was the subject of a traumatic neurosis, the result of a blow upon the head, symptoms of motor paresis, various paræsthesias, diminution of patellar reflex, staggering gait, diminution of sexual power, loss of memory and mental power, together with a number of minor phenomena. After several months the case terminated in death, and at the autopsy there were noted marked sclerotic changes in the cerebral mass, as well as in the spinal tracts, notably in the posterior columns. That such marked alterations should quickly lead to death, and should arrive thus at recognition at the autopsy, is not in the least surprising; yet the case may be regarded as an exceptional one in the degree of severity of the changes, and it should be no more surprising that the probably more minute and more intimate changes of the ordinary case should not lead to a fatal termination and subsequent discovery. That these possible changes are of the same inherent nature, degenerative and sclerotic, is held by a no inconsiderable proportion of authorities, the genesis of the lesions depending perhaps upon primary vaso-motor and vascular changes. It was first believed that the symptoms were dependent upon actual changes, seated in the spinal cord or in its adjacent structures and the emerging nerves (Erichsen, Concussion of the Spine, etc., London, 1882); it must be acknowledged, however, that there is a strong probability of the association of cortical cerebral fault, inasmuch as otherwise there can be no proper solution of the existence of many of the allied symptoms. The assertions of Charcot and his followers that the entire symptom complex is entirely without foundation, because of the ability in some instances to produce like alterations by hypnotic suggestion, cannot be accepted as final, especially as there is no proof that such influence is without any constitutional alteration if continued in its practice.

Whatever these lesions may be, it is as logical to ascribe them to an, as yet, undescribed alteration of structure, as it is to ascribe mental function to a hitherto unproved seat in the brain; and the reality of these lesions is argued by no stronger evidence than the reality of the symptoms. In this particular relation, the above case may be referred to with some confidence, in that there exist, apart from the obviously subjective symptoms, a number of signs which possess real objective value. Such, for example, is the difference in surface temperature noted between the two sides of the body; the difference in measurement of the limbs, the contraction of the field of vision, the muscular spasm upon exciting the tender foci along the spine by means of heat. Moreover, it is little likely that with a purely functional basis there should have escaped from the anæsthetic area the patch of surface corresponding to the portion enervated by the two lower sacral nerves. Where such well defined phenomena can be detected, and are open to corroboration at subsequent examinations, there can be little question as to the existence of intentional simulation.

In the case of H—, were one to have an opportunity of examination of the nervous system, it is probable that any alterations which might be present would be found not simply in the spinal cord, its emergent nerves, or membranes, but, in view of the one-sided arrangement of the symptoms, in the madulla, or above it. That the trifacial nerve, either in its nucleus or cortically, is involved, is well demonstrated in the sensorio-sensory changes of the head. It is possible to account for the deafness by involvement of the auditory nerve, and that the facial nerve is without implication is not proved by the absence of distinct signs of facial paralysis, since the degree of impairment is apparently slight in all the motor nerves.

However, over it all there must be acknowledged the existence of an overwhelming stratum of hysteria, of a psychosis, and until the pathological foundation of that state may be asserted, there will necessarily remain a great element of the problematic in the discussions as to the so-called railway spine.

